# Does White Clover (*Trifolium repens*) Abundance in Temperate Pastures Determine *Sitona obsoletus* (Coleoptera: Curculionidae) Larval Populations?

**DOI:** 10.3389/fpls.2016.01397

**Published:** 2016-09-16

**Authors:** Mark R. McNeill, Chikako van Koten, Vanessa M. Cave, David Chapman, Hamish Hodgson

**Affiliations:** ^1^AgResearchChristchurch, New Zealand; ^2^AgResearchHamilton, New Zealand; ^3^DairyNZ, Lincoln UniversityLincoln, New Zealand

**Keywords:** insect pest management, biological control, dairy pasture, pasture persistence, *Microctonus aethiopoides*

## Abstract

To determine if host plant abundance determined the size of clover root weevil (CRW) *Sitona obsoletus* larval populations, a study was conducted over 4 years in plots sown in ryegrass (*Lolium perenne*) (cv. Nui) sown at either 6 or 30 kg/ha and white clover (*Trifolium repens*) sown at a uniform rate of 8 kg/ha. This provided a range of % white clover content to investigate CRW population establishment and impacts on white clover survival. Larval sampling was carried out in spring (October) when larval densities are near their spring peak at Lincoln (Canterbury, New Zealand) with % clover measured in autumn (April) and spring (September) of each year. Overall, mean larval densities measured in spring 2012–2015 were 310, 38, 59, and 31 larvae m^-2^, respectively. There was a significant decline in larval populations between 2012 and 2013, but spring populations were relatively uniform thereafter. The mean % white clover measured in autumns of 2012 to 2015 was 17, 10, 3, and 11%, respectively. In comparison, mean spring % white clover from 2012 to 2015, averaged c. 5% each year. Analysis relating spring (October) larval populations to % white clover measured in each plot in autumn (April) found the 2012 larval population to be statistically significantly larger in the ryegrass 6 kg/ha plots than 30 kg/ha plots. Thereafter, sowing rate had no significant effect on larval populations. From 2013 to 2015, spring larval populations had a negative relationship with the previous autumn % white clover with the relationship highly significant for the 2014 data. When CRW larval populations in spring 2013 to 2015 were predicted from the 2013 to 2015 autumn % white clover, respectively, based on their positive relationship in 2012, the predicted densities were substantially larger than those observed. Conversely, when 2015 spring larval data and % clover was regressed against 2012–2014 larval populations, observed densities tended to be higher than predicted, but the numbers came closer to predicted for the 2013 and 2014 populations. These differences are attributed to a CRW population decline that was not accounted by % white clover changes, the CRW decline most likely due to biological control by the Braconid endoparasitoid *Microctonus aethiopoides*, which showed incremental increases in parasitism between 2012 and 2015, which in 2015 averaged 93%.

## Introduction

Weevils belonging to the genus *Sitona* include a number of species that are recognized pests including *Sitona lineatus* L. (Cantot, 1989; Lohaus and Vidal, 2010), *S. discoideus* Gyllenhal (Aeschlimann, 1978; Goldson et al., 1988), *S. hispidulus* F. (Quinn and Hower, 1986; Dintenfass and Brown, 1988) and *S. obsoletus* Gmelin (Murray and Clements, 1998; Gerard et al., 2007). *S. obsoletus* (formerly described as *S. lepidus* and *S. flavescens*), is a Palaearctic species first detected in the North Island of New Zealand in 1996 (Barratt et al., 1996). It was first discovered in the South Island of New Zealand in 2006, with discrete populations located near Richmond, Rai Valley and Christchurch (Phillips et al., 2007). The weevil shows a strong preference for white clover (*Trifolium repens* L.), although overseas it has also been considered a pest of red clover (*T. pratense* L.) (Brudea, 1982; Murray and Clements, 1994). Worldwide, white clover is recognized as a valuable component of pastures because of its ability to fix nitrogen and provide quality feed for improved animal production. In New Zealand, ryegrass (*Lolium* spp. L.) and white clover are the predominant plant species in improved grasslands (Woodward et al., 2003; Tozer et al., 2014), so the potential impact from *S. obsoletus* was considerable.

*Sitona obsoletus* is found almost all year round and depending on season, both adults and larvae can be present in pasture where white clover is growing. Under severe larval infestations, a decrease in percent foliar N levels in spring can occur, with subsequent reductions in herbage dry matter (DM) yield levels (Gerard, 2002). Under severe infestations, and in combination with livestock herbivory, plant stress or poor fertility, white clover plants can disappear from the sward. Although this is often temporary, it does have implications for pasture productivity and cost to the farmer through a reliance on artificial N to compensate for the loss of white clover. New generation adults commence emergence in spring (November), with peak emergence occurring in the following 4–6 weeks. Following emergence, adults feed extensively on the foliage, and can be damaging to both seedling clover and plants in mature stands, particularly when populations are high in early-mid-summer. During this period, flight muscles and the reproductive system are maturing. Following mating, females exhibit reproductive maturation with egg laying commencing in mid-summer, and continuing through winter into spring. The percentage of reproductively mature females is highest over winter–spring with eggs laid indiscriminately in the foliage. Eggs accumulate in the litter, with larval development occurring at temperatures above 9.8°C (Arbab and McNeill, 2011). First instar larvae emerge and burrow down into the soil to locate the root system. There are five larval instars, with the first instar dependent on root nodules for establishment and survival (Gerard, 2001) and inoculated nodules with viable rhizobia preferred to uninoculated nodules (Hackell and Gerard, 2004). Later instars feed on the general root system including the stolons. There are two larval generations per year in the Canterbury region of New Zealand, with first major peak occurring in October–November (spring), and a second smaller peak occurring in April–May (autumn) (M. R. McNeill, unpublished data).

The arrival of clover root weevil (CRW) and its natural and anthropogenic-assisted spread across New Zealand, coincided with farmer reports of white clover production and consequent animal production declines as population numbers built up in newly colonized regions (e.g., McNeill et al., 2012). This was also confirmed by small plot study that showed up to a 35% decline in DM production under a modest population of c. 300 larvae m^-2^ (Gerard et al., 2007). Management recommendations to minimize impacts included increased applications of artificial nitrogen, especially in spring, pasture management to encourage white clover production, and a prolonged fallow or crop to eliminate larval populations prior to resowing of new pastures. As part of the management program to control *S. obsoletus*, the Braconid endoparasitoid *Microctonus aethiopoides*, was introduced in 2006 (Gerard et al., 2006; Phillips et al., 2007). Originally from Ireland, the parasitoid attacks the adult weevil, with parasitism resulting in sterilization and the eventual death of the weevil upon emergence of the parasitoid larvae. For female CRW, parasitism prevents further egg laying, with a subsequent impact on the number of larvae that establish. The parasitoid is able to complete 3–4 generations per year, attacks multiple weevils and has a winter diapause, occurring as a first instar larva inside the adult weevil. As of June 2016, CRW is widely distributed across New Zealand pasture containing white clover, and depending on season, either adults or larvae or both stages occur concurrently.

The importance of root nodules to *Sitona* spp. larval development has been shown in several contributions (e.g., Danthanarayana, 1967; Quinn and Hower, 1986; Wolfson, 1987; Murray and Clements, 1998; Gerard, 2001; Lohaus and Vidal, 2010), although the relationship was less clear for *S. discoideus* first instar survival (Aeschlimann, 1986). The result of nodule feeding and destruction of the root system, is that the plant can become stressed causing overcompensation in nodule production (Quinn and Hall, 1992), reduction in leaf and root N (Murray et al., 2002), modification to C:N ratios (Murray et al., 1996) and overall reductions in DM production (Dintenfass and Brown, 1988; Goldson et al., 1988). As CRW egg deposition and subsequent larval establishment is a function of time and place, female CRW have an important role in distributing the eggs in an environment that is conducive to larval survival (Johnson et al., 2006). For this reason, the number of adults and resultant egg laying effort could be expected to be higher in swards supporting a greater white clover content compared with pastures with a low white clover content. Gerard et al. (2007) reported that plots with good clover cover had more than twice the number of larvae m^-2^ compared to plots with low % clover. Therefore, it was hypothesized that the density of CRW larvae is a function of white clover content, with % white clover an indicator of root resources, especially root nodules. In other words, high CRW larval populations occur in swards with a high % white clover, conversely low populations will occur in swards with a low % white clover. In addition, it was considered that the % white clover measured in autumn, at the start of the ovipositional effort, would be a primary determinant of subsequent CRW larval populations in spring.

The establishment of a DairyNZ field trial in March 2010, to investigate ryegrass persistence under a range of sowing rates also provided an opportunity to measure CRW larval densities each spring in order to assess any impact of CRW on white clover production. In addition, it offered an opportunity to see if the presence of *M. aethiopoides* had a long term impact on CRW larval populations. CRW adults were first detected on the Lincoln University Research Dairy Farm in late 2009. The Irish *M. aethiopoides* was first recorded in CRW collected from a paddock on the farm on 14 March 2011 with a parasitism rate of 46%. This high level of parasitism indicated that the parasitoid was possibly already established at the beginning of 2011.

## Materials and Methods

### Research Site

The research was undertaken on plots within the Longitudinal Persistence Experiment (Sub-project 3 of FD1004) run by DairyNZ (Lee et al., 2016). The experiment was on the Lincoln University Research Dairy Farm (S43.636721, E172.460865), with research investigating the effect of ryegrass seed rates on plant size, competitive ability and persistence, as well as establishment and subsequent presence of white clover in the sward. Ryegrass and clover were drilled into cultivated seedbed between 30 March – 4 April 2011. The DairyNZ experiment comprised four ryegrass cultivars [cv. Alto AR37 (diploid), cv. Commando AR37 (diploid), cv. Halo AR37 (tetraploid) and cv. Nui wild-type endophyte (diploid)]. There were five ryegrass seeding rates for each cultivar: 6, 12, 18, 24, and 30 kg/ha of Ultrastrike^®^ – insecticide treated seed. All plots were drilled with 8 kg/ha of Superstrike^®^ – insecticide treated white clover seed (cv. Tribute) (equivalent of 5 kg/ha of bare seed). Treatments were arranged in a randomized split block design with five block replicates. The site was irrigated and grazed by dairy cows (see grazing details below).

In the study reported here, CRW larval and % clover measurements were restricted to the ryegrass cv. Nui plots sown at 6 and 30 kg/ha.

### Sampling CRW Larvae and Adults

Soil cores (10 cm diameter by 14 cm deep) were taken from each of the five replicates of both 6 and 30 kg/ha ryegrass-white clover plots. In the laboratory, cores were hand sorted and second through to fourth instar larvae counted to determine the number of CRW larvae m^-2^. Because first instar larvae are difficult to locate in the soil or hidden in the clover root nodules these were not counted. Therefore, recorded numbers are an underestimate of actual larval densities. Sampling to monitor initial establishment and build-up of larvae first occurred on 13 December 2011 approximately 8 months after the trial was sown. with a subsequent sample on 2 May 2012. On each occasion, only five cores were taken per plot. Thereafter, sampling occurred 25 October 2012, 24 October 2013, 22 October 2014, and 20 October 2015 with 10 cores taken from each plot.

Sampling to detect the presence of adult CRW was first carried out on 13 December 2011. Sampling on this occasion was undertaken using a vacuum cleaner to remove weevils from 0.25 m^-2^ quadrats, with five quadrats per replicate, giving 50 samples in all. This method provided an estimate of ground density at the start of immigration by spring-emerged adults. Thereafter, adults were sampled in May or July each year, using a modified blower vac (Echo ES-2400, 24 cc, Kioritz Corporation, Tokyo) to collect the weevils from the foliage and litter. CRW adults were extracted from the sample, counted and dissected under a binocular microscope to determine reproductive condition and parasitism status. The period May to July provides an accurate fix on parasitism, as *M. aethiopoides* enters diapause in late autumn, overwintering as first instar larvae inside the weevil. The blower vac was specifically utilized for collecting sufficient numbers of weevils for dissection and not to ascertain adult densities.

### White Clover Measurements

Details on the collection and assessment of botanical composition are detailed in Lee et al. (2016). Briefly, representative samples of herbage from each sub-plot were collected the day before grazing in autumn (April) and late spring (August/September). Cuts were taken using hand shears, returned to the laboratory and a sub-sample dissected into the following categories: perennial ryegrass leaf, perennial ryegrass reproductive stem (including seed-head and flowers), white clover, unsown species and dead material. The percentage white clover was measured on 16 April 2012, 22 April 2013, 14 April 2014, and 31 March 2015 (autumn) and then 27 August 2012 (late winter), 23 September 2013, 23 September 2014, and 27 October 2015 (spring).

### Fertilizer and Grazing Management

Maintenance fertilizer was applied to the Canterbury site in June 2012 (61, 38, 74, and 135 kg ha^-1^ of P, K, S, and Ca; Lee et al., 2016). Nitrogen (N) fertilizer was applied as urea with total yearly N applications across the 4 years being 198 kg ha^-1^ (2011/12), 264 kg ha^-1^ (2012/13), 280 kg ha^-1^ (2013/14), and 225 kg ha^-1^ (2014/15), respectively, spread over three to six applications. Plots were rotationally stocked by dairy cows, grazed at the same time to a desired residual and as required, mown post-grazing to maintain a uniform pasture (Lee et al., 2016).

### Data Analysis

#### Larval Density by Year

This analysis compared the larval populations in October from 2012 to 2015. Analysis took a generalized estimating equations (GEE) approach, using a generalized linear model assuming group-specific negative binomial distributions through log link function. The groups were defined by a single factor Year (four levels: October 2012 to October 2015). The GEE analysis also took account of potential correlation among larval numbers in soil cores taken from each individual plot in each year.

#### Percentage White Clover by Year

The % white clover values were analyzed and compared between the 4 years; 2012 to 2015, using analysis of variance (ANOVA) with a single factor: Year (four levels: October 2012 to October 2015). The ANOVA was applied to % clover in autumn and spring, respectively.

#### Larval Density and % White Clover within a Year

As CRW larvae require white clover to survive, it was hypothesized that the percentage white clover measured in the pasture was an indicator of the size of the CRW larval populations (e.g., high clover content indicated a high larval population). This was based on the premise that CRW adult populations would colonize swards with high white clover content. Furthermore, % white clover measured in autumn would reflect both the availability of plant material for adult CRW and nodule resources for the putative larval population going into winter. Therefore, low % white clover in autumn would be expected to result in low CRW larval populations in spring. An assessment using spring measurements of % white clover was also carried out to examine the relationship between the larval populations and % white clover in spring. In order to test this hypothesis, larval populations measured each October 2012 to 2015, irrespective of ryegrass sowing rate, were analyzed using GEE, against % white clover measured in autumn (April) and again in late winter and spring (August–September). Each GEE analysis used a generalized linear model assuming negative binomial distribution through log link function, and the relationship of larval population [larval density (m^-2^)] in each year to % white clover was modeled as an exponential equation:

$$Larval\,\;density\,\;(m^{-2})=127.4\times \exp\left(A+B\times \%white\,\;clover\right)$$

where 127.4 was a constant to convert the estimated larval density per soil core into the density m^-2^ and A and B were intercept and slope coefficient to be estimated, respectively. This GEE analysis also took account of potential correlation among larval numbers in soil cores taken from each individual plot in each year. All GEE analyses and ANOVAs were carried out using SAS (version 9.3).

#### Larval Density and % White Clover between Years

Within any year the range of % white clover was small, however, between years the range was much greater. Therefore, in order to further investigate whether there was any relationship between spring larval density (m^-2^) with % white clover measured in autumn or spring, a repeated measures analysis of the per plot data across all 4 years was performed. The correlation between mean larval densities in an individual plot over time was modeled using an autoregressive model of order 1. Additional uniform correlation within plots was also allowed for. Larval density was log_10_+10 transformed prior to analysis to stabilize the variance. Ten was added prior to log_10_ transformation to accommodate the presence of zero densities observed in 2015. The model was fitted using restricted maximum likelihood (REML) in Genstat (version 17).

A simple linear regression model was also fitted to the 2012 log_10_ (spring larvae) data, with either the % clover in spring, summer or autumn as the explanatory variable. From the fitted model, the log_10_ (spring larvae) in 2013, 2014, and 2015 was predicted. Similarly, using 2015 spring larval data with % clover in either in spring, summer or autumn as the explanatory variable, the 2012, 2013, and 2014 larval populations were predicted. However, in contrast to the 2012 larval data, the 2015 spring larvae data didn’t require transformation prior to analysis in order to linearize the relationship.

## Results

### CRW Larval Populations

There were no CRW larvae found in the cores taken on 13 December 2011, which is consistent with our understanding that colonization of the newly established plots by spring emerged CRW adults would have only just begun and the lack of reproductively mature females would have meant an absence of larvae. Larval recruitment between December 2011 and May 2012 was rapid, and by 2 May 2012, mean (±SEM) larval populations in the 6 and 30 kg/ha plots were 234 ± 86.6 and 246 ± 124.9 larvae m^-2^, respectively, and not significantly different (*P* = 0.887).

Mean larval densities measured in all plots in October 2012–2015 were 310 ± 43.3, 38 ± 7.6, and 59 ± 14.131 ± 8.1 larvae m^-2^, respectively. There was a significant decline in larval populations between 2012 and 2013 (*P* < 0.001), thereafter larval densities between the October 2013–2015 samples were not significantly different (*P* > 0.05).

**FIGURE 1:**
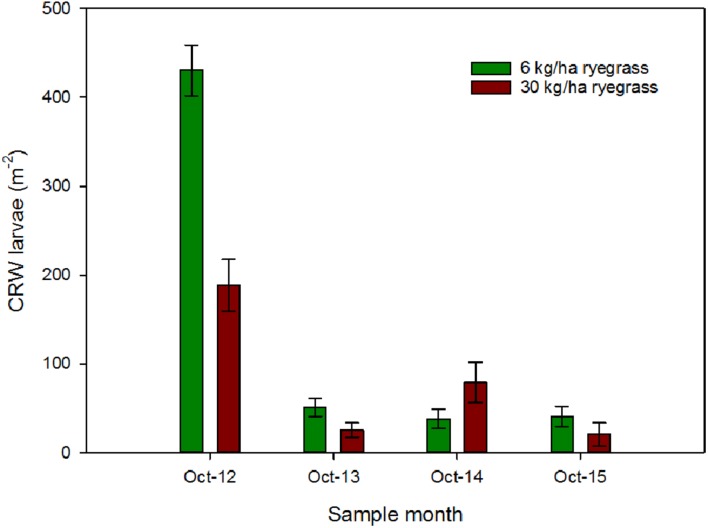
**CRW larval densities (±SEM) measured in ryegrass/white clover swards consisting of ryegrass sown at 6 or 30 kg/ha and white clover sown at 8 kg/ha**.

Ryegrass sowing rate had a significant influence on the larval populations in October 2012, with mean densities of 431 ± 28.7 and 188 ± 28.9 larvae m^-2^ in the 6 and 30 kg/ha plots, respectively (*P* < 0.001; **Figure 1**). Sampling in October 2013, found a significant decline in larval populations (**Figure 1**) at both sowing rates compared to 2012, but sowing rate had no significant effect on larval densities (*P* = 0.064), a result that was repeated in the following 2 years (**Figure 1**).

### CRW Adults and Parasitism

Sampling on 13 December 2011, found a mean (±SEM) of only 1.0 ± 0.33 adults m^-2^ with all eight individuals immature and unparasitised. Subsequent dissection of weevils collected in May–July from 2012 to 2015, found parasitism by *M. aethiopoides* was high to very high (38–95%) and generally increased over the study period. Dissection of CRW adults collected on 14 May 2012 and on 16 May 2013, showed high rates of parasitism of 79% (*n* = 39) and 84% (31), respectively. Sampling on 01 July 2014, found an average parasitism rate of 58% (range 38–94%). However, in 2015, the mean parasitism rate across all plots was 93%, indicating both a significant increase in overall parasitism compared to 2014 and subsequent impact on CRW oviposition potential.

### Percentage White Clover by Year

The mean % white clover measured in autumn of 2012 to 2015 was 17 ± 2.2, 10 ± 2.3, 3 ± 2.3 and 11 ± 2.3%, respectively. The declines between 2012 and 2013, and between 2013 and 2014 were both significant (*P* = 0.043 and 0.025, respectively). By comparison, there was a significant increase between 2014 and 2015 (*P* = 0.016).

The mean % white clover measured in spring of 2012, 2013, and 2014 was relatively constant at c. 5 ± 1.2 for each of the 3 years but in 2015 averaged 6.4 ± 1.2, the slight increase possibly related to the later sampling and the onset of spring clover growth.

**FIGURE 2:**
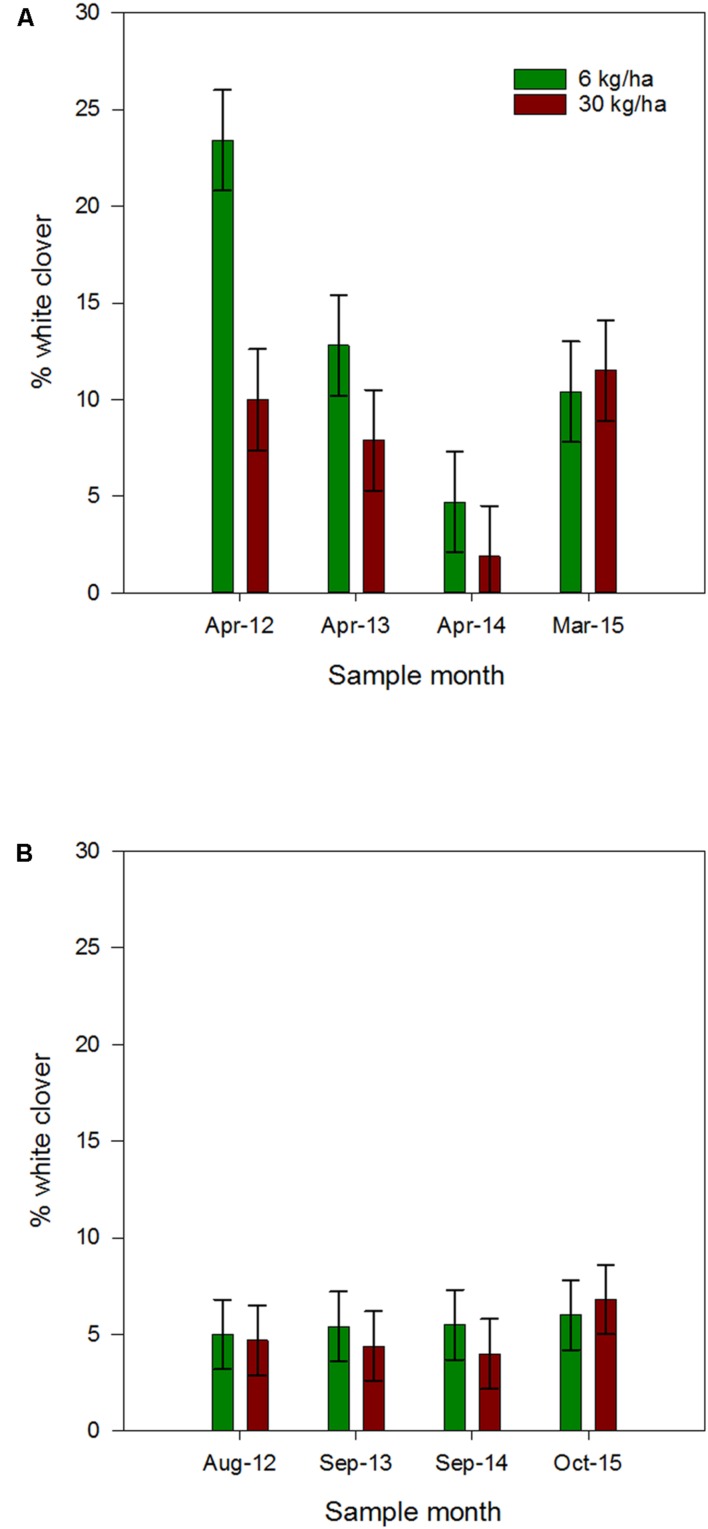
**Mean autumn **(A)** and spring **(B)** % white clover (±SEM) measured in plots containing ryegrass sown at 6 or 30 kg/ha and white clover sown at 8 kg/ha.** Autumn values indicated in **(B)** for comparison.

Ryegrass sowing rate had a significant influence on the % white clover measured in autumn, with white clover content higher in plots sown with ryegrass at 6 kg/ha compared to the 30 kg/ha sowing rate (**Figure 2A**). This was most notable in the April 2012 assessment, where the mean % white clover measured in the 6 kg/ha and 30 kg/ha plots was 23.4 ± 2.7 and 10 ± 2.7%, respectively, and significantly different (*P* = 0.002). Thereafter, the % white clover was not significantly different at the two ryegrass sowing rates. Overall, at the 6 kg/ha ryegrass sowing rate, autumn % white clover showed a significant decline each year, while for the 30 kg/ha sowing rate the decline was only significant between April 2012 and April 2014 (*P* = 0.043).

By comparison, the mean % white clover measured in late winter and spring (August 2012, September 2013–2015), across both sowing rates showed no significant difference between years or ryegrass sowing rate (**Figure 2B**).

## CRW Larval Populations and Interaction with % White Clover

**FIGURE 3:**
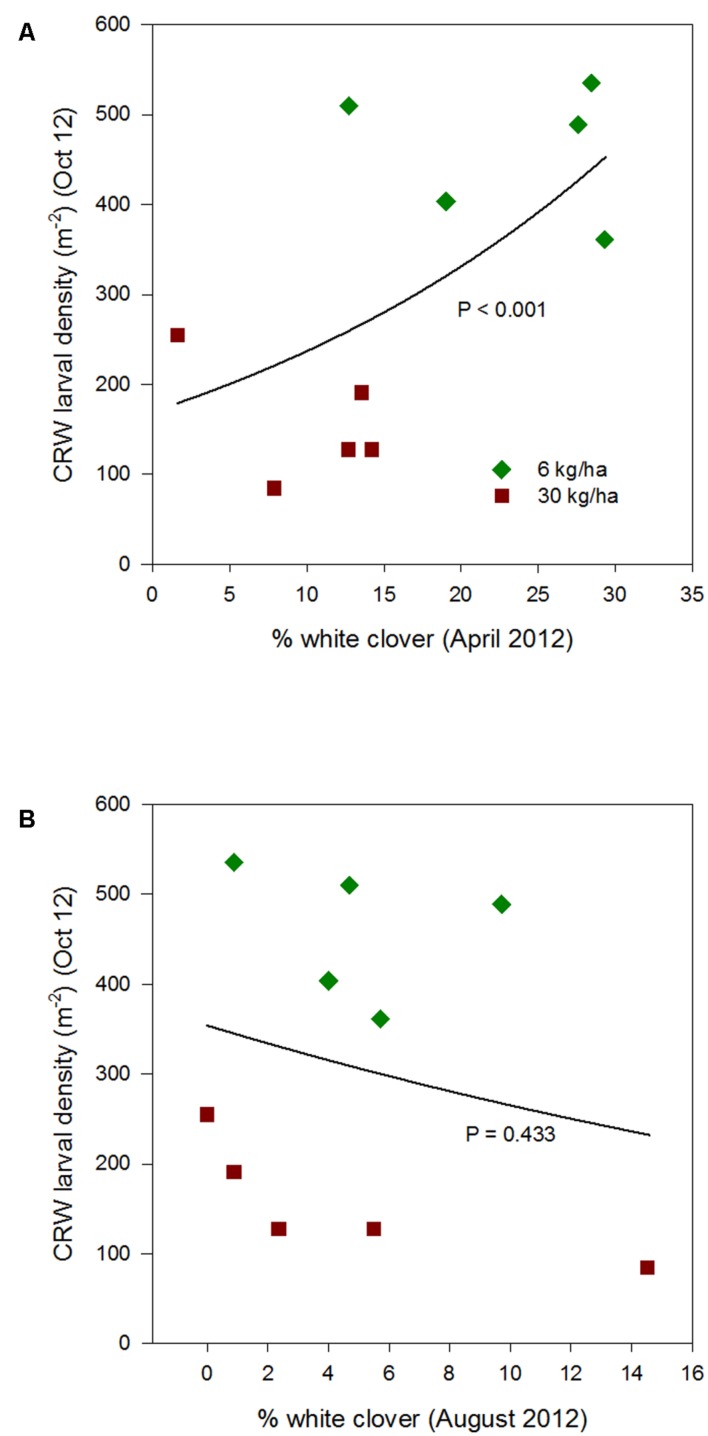
**Clover root weevil larval densities measured in October 2012 in relation to % white clover measured in April 2012 **(A)** and August 2012 **(B)**, from ryegrass plots (cv. Nui) sown at 6 and 30 kg/ha (solid line shows the estimated relationship across all plots, irrespective of ryegrass sowing rate)**.

**FIGURE 4:**
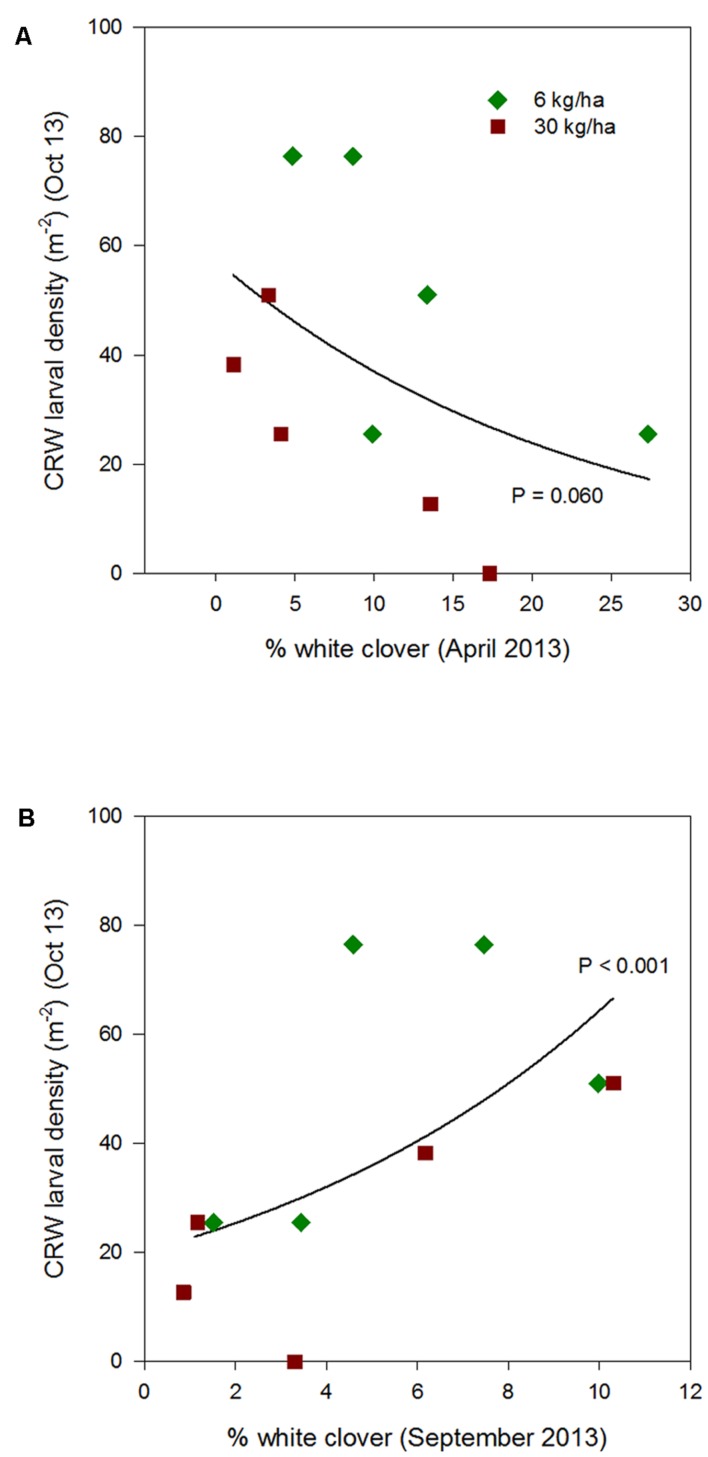
**Clover root weevil larval densities measured in October 2013 in relation to % white clover measured in April 2013 **(A)** and September 2013 **(B)**, from ryegrass plots (cv. Nui) sown at 6 and 30 kg/ha (solid line shows the estimated relationship across all plots, irrespective of ryegrass sowing rate)**.

**FIGURE 5:**
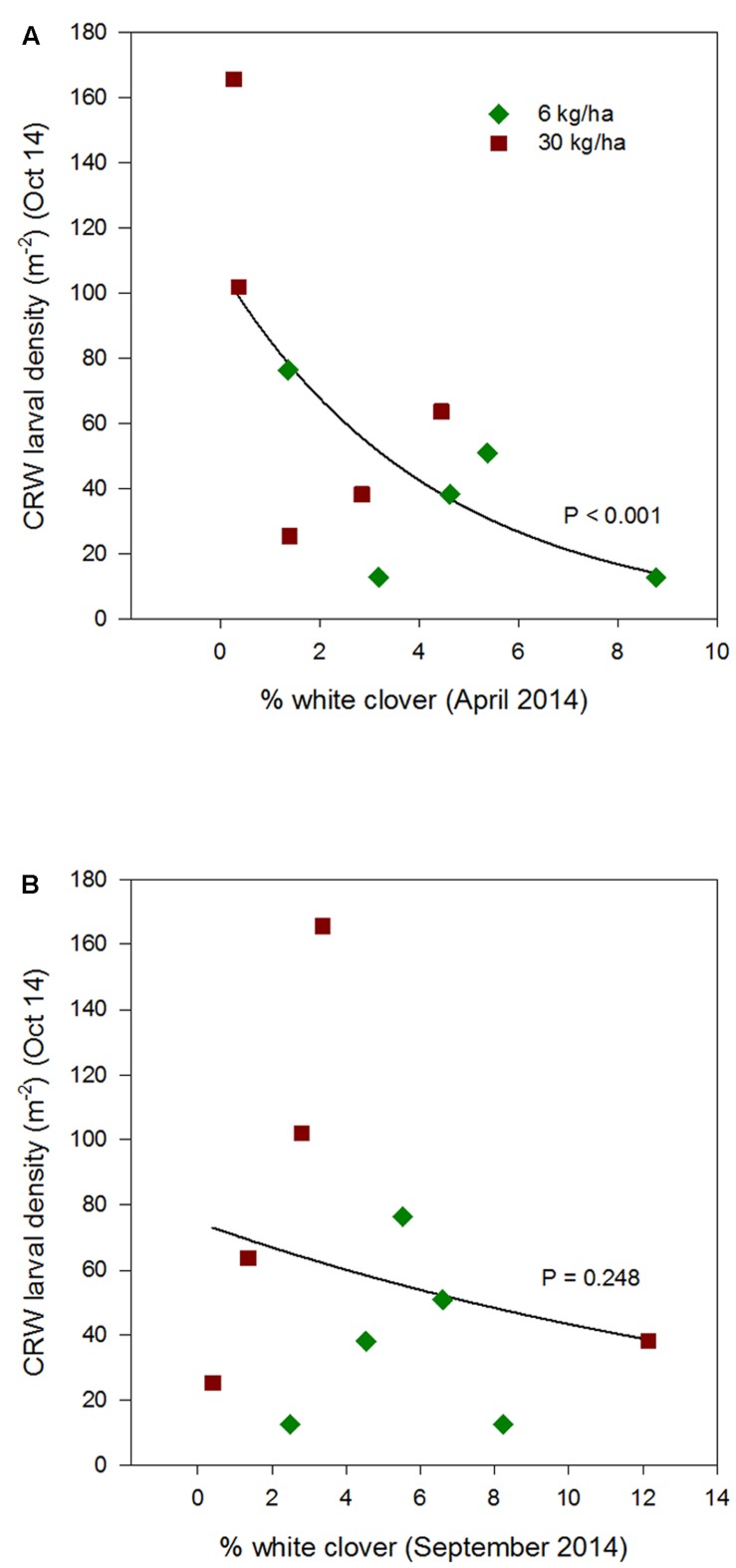
**Clover root weevil larval densities measured in October 2014 in relation to % white clover measured in April 2014 **(A)** and September 2014 **(B)**, from ryegrass plots (cv. Nui) sown at 6 and 30 kg/ha (solid line shows the estimated relationship across all plots, irrespective of ryegrass sowing rate)**.

**FIGURE 6:**
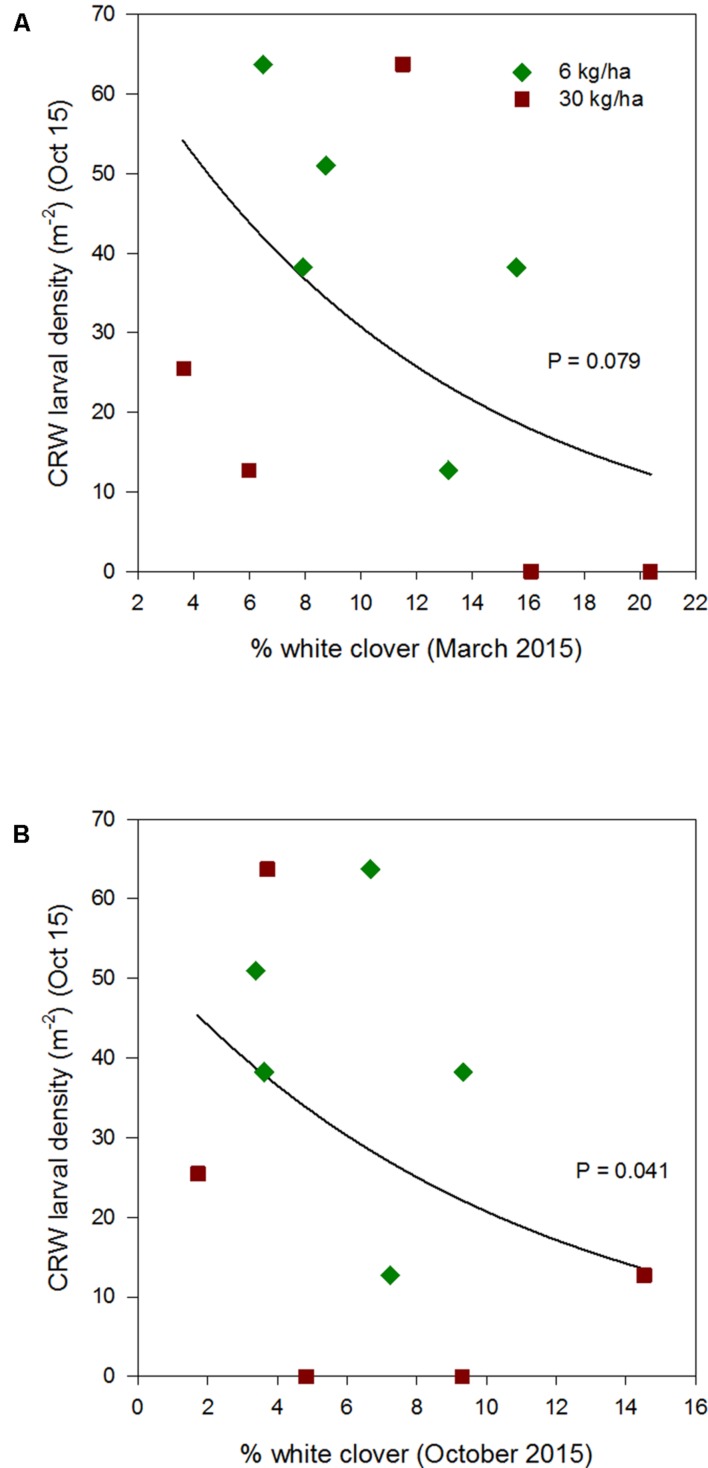
**Clover root weevil larval densities measured in October 2015 in relation to % white clover measured in March 2015 **(A)** and October 2015 **(B)**, from ryegrass plots (cv. Nui) sown at 6 and 30 kg/ha (solid line shows the estimated relationship across all plots, irrespective of ryegrass sowing rate)**.

From 2012 to 2015, CRW larval populations showed a relationship with % white clover that varied across years and by season (**Figures 3**–**6**; **Table 1**).

**Table 1:** Equations describing the relationship between CRW larval density and % white clover (% wc) measured from 2012 to 2015.

Larval sample month	% clover sample	Estimated relationship	*P*-value
October 2012	April 2012	Larval density (m^-2^) = 127.4 × Exp (0.287 + 0.0334^∗^% wc)	<0.001
	August 2012	Larval density (m^-2^) = 127.4 × Exp (1.0213 - 0.0289^∗^% wc)	0.433
October 2013	April 2013	Larval density (m^-2^) = 127.4 × Exp (-0.799 - 0.0438^∗^% wc)	0.060
	September 2013	Larval density (m^-2^) = 127.4 × Exp (-1.844 + 0.1160^∗^% wc)	<0.001
October 2014	April 2014	Larval density (m^-2^) = 127.4 × Exp (-0.168 - 0.2321^∗^% wc)	<0.001
	September 2014	Larval density (m^-2^) = 127.4 × Exp (-0.535 - 0.0541^∗^% wc)	0.248
October 2015	March 2015	Larval density (m^-2^) = 127.4 × Exp (-0.536 - 0.0886^∗^% wc)	0.079
	October 2015	Larval density (m^-2^) = 127.4 × Exp (-0.871 - 0.0945^∗^% wc)	0.041


### Interaction with % White Clover Measured in Autumn

Larval populations measured in October 2012 were significantly higher in plots where ryegrass had been sown at 6 kg/ha compared to plots sown at 30 kg/ha (**Figure 1**). This corresponded with the significantly larger mean % white clover in April 2012 in the 6 kg/ha plots (**Figure 2**). This sowing rate group difference also led to a statistically significant positive correlation with the % white clover overall (*P* < 0.001; **Figure 3A**; **Table 1**).

Larval populations measured in October 2013, 2014, and 2015, had a negative relationships with the autumn % white clover (**Figures 4A**, **5A**, and **6A**, respectively, **Table 1**) with the relationship highly significant for the 2014 data (*P* < 0.001; **Figure 5A**).

### Interaction with Percentage White Clover Measured in Spring

The relationship of October CRW larval populations with % white clover measured in spring (late August 2012, September 2013 to 2015) was also mixed. There was no significant larvae – % white clover interaction measured in August 2012 (*P* = 0.433; **Figure 3B**; **Table 1**). Conversely, in 2013, the October larval population had a significant positive relationship with % white clover measured in September (*P* < 0.001; **Figure 4B**; **Table 1**). In 2014, there was slightly negative, but not significant relationship between the October larval population and % white clover measured in September (*P* = 0.248; **Figure 5B**; **Table 1**). For 2015, the relationship was again negative and significant (*P* = 0.041; **Figure 6B**; **Table 1**).

### Larval Density and % White Clover across Years

**FIGURE 7:**
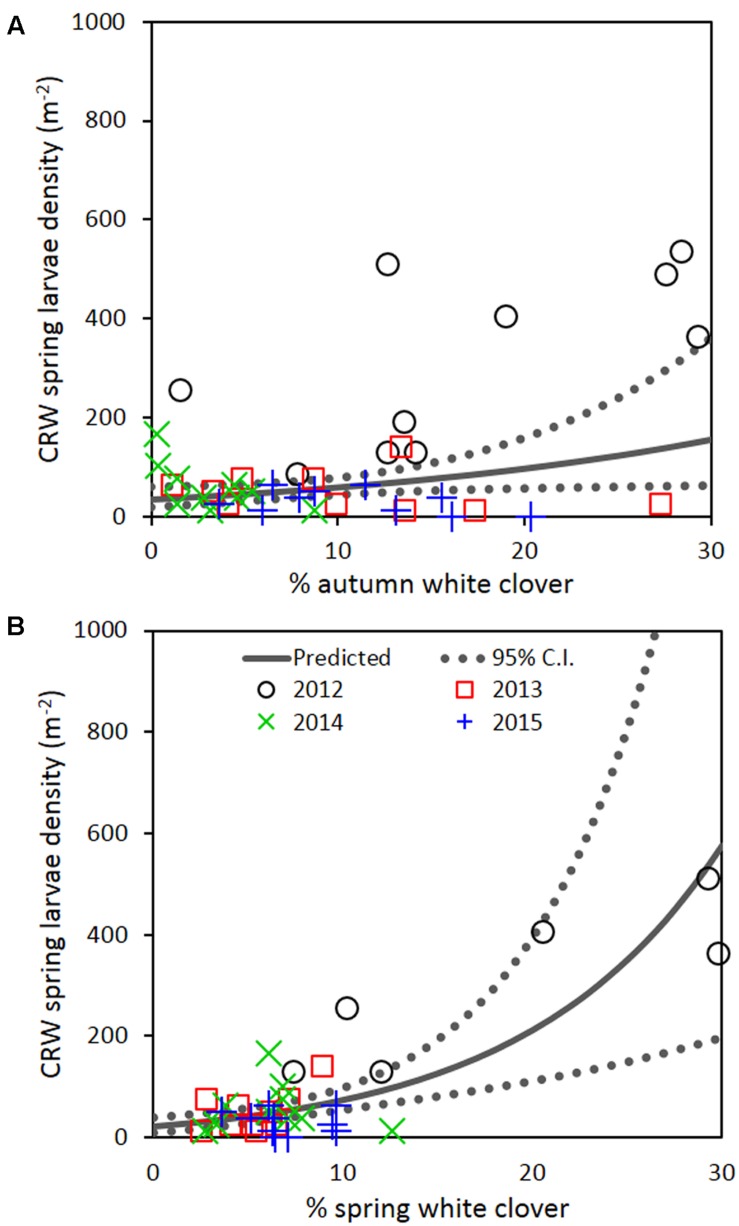
**Clover root weevil larval densities measured in spring in relation to % white clover measured in **(A)** autumn and **(B)** spring of 2012–2015.** The solid line shows predicted density from the repeated measures analysis, with the dotted lines representing 95% confidence intervals of the predicted.

Repeated measures analysis across all 4 years detected a significant positive relationship between spring CRW larval density with % white clover measured in autumn (*P* = 0.030; **Figure 7A**; **Table 2**) and spring (*P* < 0.001; **Figure 7B**; **Table 2**). The apparent contradiction with the year results may be due to the small range of % white clover values within years and/or the high leverage data from 2012 before the CRW population collapsed had on the analysis (**Figure 7**). If data from 2012 is omitted, there is no longer evidence of a positive relationship.

**Table 2:** Repeated measures analysis of transformed spring CRW larval density against % white clover measured from 2012 to 2015.

Season	Slope estimate for % white clover	SE of slope	df	*P*-value
Autumn	0.0189	0.0083	36	0.030
Spring	0.0422	0.0100	37	<0.001


If the decline of CRW larvae from 2012 to 2013 followed the decline of % white clover between autumn of 2012 and 2013, it is not unreasonable to consider that the spring larval population had a positive relationship with white clover coverage in the previous autumn (i.e., higher the white clover coverage in autumn, the larger the larval population in spring). However, between 2013 and 2014, the larval population did not follow the further autumn % white clover decline and instead, slightly increased, although the increase was not statistically significant. In addition, the spring larval populations in 2013, 2014, and 2015 had rather negative relationships with the previous autumn clover coverage, with most of these negative relationships found to be statistically significant (**Table 1**). These results are contradictory to a positive correlation expected from the hypothesis.

## Discussion

This study observed a rapid colonization of newly established white clover pastures by CRW, which went from no larvae in December 2011 but by May 2012 had increased to a mean of 240 larvae m^-2^. In those first 18 months following establishment, colonization was greater where there was more clover in the sward and supported the hypothesis that clover content supports higher larval densities. Once the CRW population was established, the autumn clover content declined sharply which supports the idea that the clover decline was driven by the impact of CRW. Thereafter, there was no consistent relationship between spring larval population and % white clover in autumn, or % white clover in spring.

**FIGURE 8:**
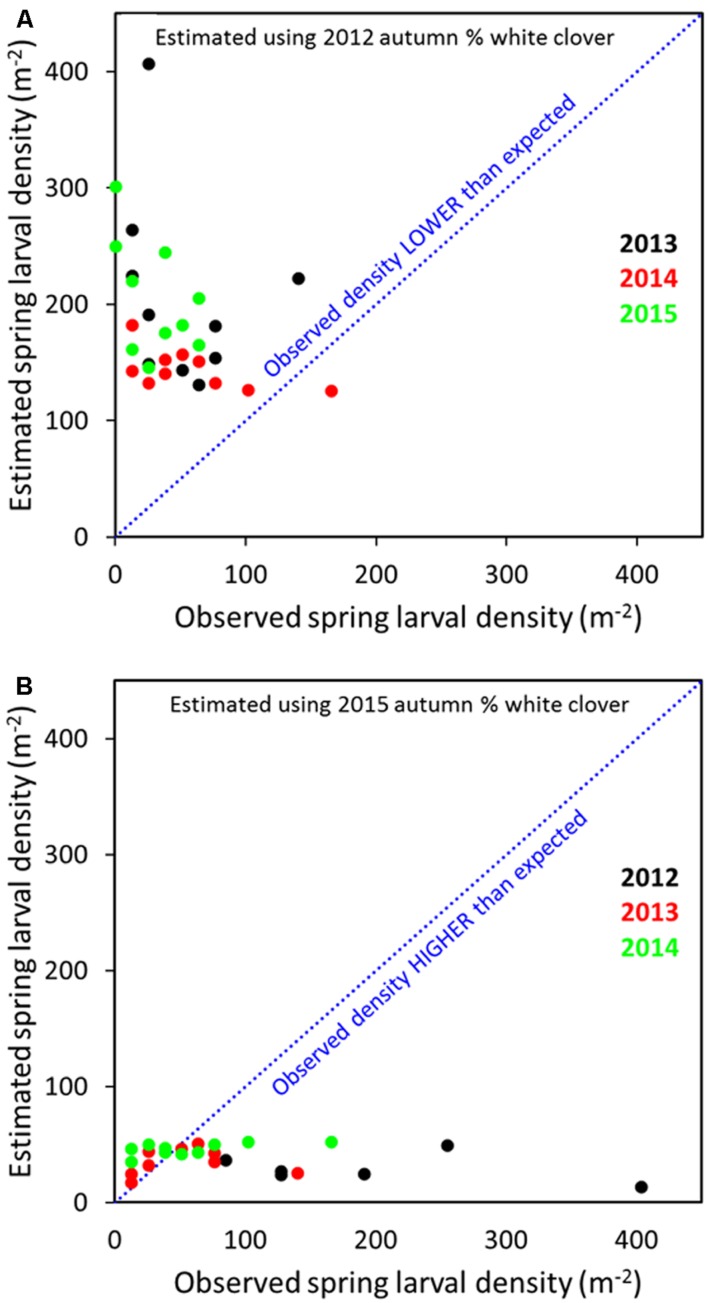
**Predicted and observed *S. obsoletus* larval densities for 2013–2015 populations based on 2012 log_10_ spring larvae data, with % autumn white clover as the explanatory variable **(A)** and 2012–2014 larval populations based on 2015 spring larval data with % autumn white clover as the explanatory variable **(B)****.

Across all years, relating the 2012 transformed spring larval data with % clover in spring, summer or autumn 2012 indicated that predicted populations for 2013 to 2015 were larger than observed (**Figure 8A**; autumn data only). Conversely, when 2015 spring larval data and % clover was regressed against 2012–2014 larval populations, observed densities tended to be higher than predicted, but the numbers came closer to predicted for the 2013 and 2014 populations (**Figure 8B**). The presence of the *M. aethiopoides* and its impact on CRW egg laying is considered an important factor in reducing larval populations. Based on dissection data, high levels of parasitism were observed on the site. This meant a significant decline in egg laying potential by parasitized female CRW, which consequently reduced larval recruitment. Root nodule availability is a significant driver to CRW first instar establishment and survival (Gerard, 2001) and feature common to other *Sitona* species (e.g., Quinn and Hower, 1986; Goldson et al., 1988; Lohaus and Vidal, 2010), but even allowing for density dependent larval survival in relation to root nodule availability (e.g., Goldson et al., 1988), parasitism of adult CRW by the *M. aethiopoides* would have contributed to the overall decline in larval populations. Variation in the application of artificial nitrogen over the course of the study, as a basis for larval population changes was considered but disregarded after correlation analysis found no relationship between artificial nitrogen and larval populations (Cave, unpublished data).

Finding precedents to explain the two contradictory relationships observed at the Lincoln site (positive and negative relationships between CRW larval populations and clover coverage) is difficult. Gerard et al. (2007) found a significant positive linear relationship between mean numbers of CRW larvae present and % clover cover. This supports the positive correlation and was observed in 2012 in this study. However, in Gerard et al. (2007), measurements of the two variables (larval number and % clover cover) were taken concurrently, whereas in the work reported here there was at least a 30-day interval between the botanical and larval measurements. Whether this difference is important is debatable.

Feeding by CRW early instar larvae has been shown to have a range of impacts on infested plants, including significant reductions in leaf and root DM, the number of nodules and total N of leaf and root tissue (Murray et al., 2002). Under laboratory conditions, feeding by first instar *S. hispidulus* on *Medicago sativa* L., led to overcompensatory growth of nodules, increased N-fixation and root growth (Quinn and Hall, 1992). The rate of nodulation was highest in plants with low initial nodule biomass and lowest in plants with relatively high initial nodule biomass, suggesting that the rate of compensatory nodulation may increase as feeding by nodule herbivores increases. However, at some point repeated denodulation will lead to significant damage to the root system and yield losses (e.g., Goldson et al., 1988). Quinn and Hower (1986) found that under field conditions first and second -instar larvae of *S. hispidulus* were correlated with small root nodules of *M sativa* and soil moisture, but not taproot biomass. For later instars, there was no correlation with nodules, with only fifth-instar larval numbers showing a correlation with taproot biomass (Quinn and Hower, 1986).

There is a paucity of studies that have described the relationship between white clover foliage and root biomass including nodule dynamics (J Crush, AgResearch, pers. comm.), particularly between above ground foliage and the root system of plants over winter in Canterbury, when white clover leaf biomass becomes reduced in size and in some cases ostensibly disappears from the sward. And the scale of this study did not allow for detailed assessment of the root biomass, particularly root nodules during sampling. For this reason, establishing the exact cause for the inverse density dependent relationship between % white clover and larval populations is problematic. Potentially, if pasture with high clover content supports a commensurate adult and hence larval population, larval competition for root resources and naturally occurring entomopathogenic diseases such as *Beauveria bassiana* (Bals.-Criv.) Vuill. and *Metarhizium anisopliae* (Metchnikoff) Sorokin, acting on populations during the winter-early spring period, may explain the observed relationships.

## Conclusion

Establishment of a white clover- ryegrass pasture, led to rapid colonization by CRW and high larval populations which showed significant declines in the following years. Parasitism of CRW adults can be considered a contributing factor to this decline in larval populations from 2013 onward. Based on % representation in plots, white clover abundance was highest in the first autumn following establishment, but significantly less in the following years. No overall relationship between CRW larval populations and % white clover was found, except when the 2012 data was included. However, within each year there were significantly relationships, often negative particularly, in 2014 and 2015 which indicated high autumn % clover had a detrimental impact on larval densities in spring, i.e., fewer spring larvae in relation to increased % clover in the previous autumn. The reasons for this are uncertain, but may be related to density dependent larval mortality due to loss of root biomass over winter. Irrespective of cause, larval populations by 2015 were significantly less than recorded in 2012, and were independent of % white clover.

## Author Contributions

MM: Developed the research concept, led and carried out the insect sampling and processing, wrote the paper. CvK and VC: Analyzed the insect and plant data and wrote the paper. DC: Developed the research concept. HH: led and carried out the plant sampling and processing.

## Conflict of Interest Statement

The authors declare that the research was conducted in the absence of any commercial or financial relationships that could be construed as a potential conflict of interest.
